# Complete Genome Sequences of 28 Lactococcal Bacteriophages Isolated from Failed Dairy Fermentation Processes

**DOI:** 10.1128/MRA.01535-19

**Published:** 2020-03-19

**Authors:** Barbara Marcelli, Anne de Jong, Thomas Janzen, Mariela Serrano, Jan Kok, Oscar P. Kuipers

**Affiliations:** a Department of Molecular Genetics, Groningen Biomolecular Sciences and Biotechnology Institute, University of Groningen, Groningen, The Netherlands; b Bacterial Physiology and Improvement, R&D Discovery, Chr. Hansen A/S, Hørsholm, Denmark; c R&D Department, CSK Food Enrichment, Wageningen, The Netherlands; DOE Joint Genome Institute

## Abstract

Lactococcus lactis is a Gram-positive lactic acid bacterium commonly used in the dairy industry for the production of fermented foods such as buttermilk and a wide variety of cheeses. Here, we report the complete genome sequences of 28 bacteriophages infecting different L. lactis industrial starter strains isolated from dairy plants throughout the world.

## ANNOUNCEMENT

Bacteriophage infection of Lactococcus lactis strains, which are used as starter cultures in dairy fermentation processes, is one of the main causes of fermentation failure and causes great economic losses for dairy industries (1, 2). Bacteriophages infecting L. lactis have been divided into 10 species (3), and those belonging to species c2, 936, or P335 are more commonly encountered in dairy plants (4, 5). However, isolates belonging to other species have also been reported to cause dairy fermentation halts (6–10). Studying lactococcal bacteriophages is of crucial importance for understanding phage-host interactions in dairy environments and for preventing the spread of infections in production lines. Here, we present the complete genome sequences of 28 lactococcal bacteriophages isolated over the past 3 decades from failed fermentations in dairy plants located throughout the world.

The bacteriophages were isolated from whey samples and plaque purified three times on their industrial lactococcal hosts at 30°C in M17 medium using the soft-agar overlay assay (11). A single plaque was finally propagated in liquid M17 medium on the same host to obtain a pure phage lysate. Phage purification was achieved by polyethylene glycol 800 (PEG 800) precipitation, and genomic DNA was extracted via phenol-chloroform purification using a previously described method (10). Samples were prepared for sequencing using a standard Illumina genomic library. The sequencing process delivered 5 million paired-end reads (2 × 150 bp) per sample. Quality control of the total sequence reads was performed with FastQC (http://www.bioinformatics.babraham.ac.uk/projects/fastqc), and the sequence reads were trimmed using Trimmomatic v0.36 (12). Genome assembly was performed using the A5-miseq pipeline with default parameters (13). The sequences of the contigs obtained were subjected to a BLAST search against the total genomes of known lactococcal strains and bacteriophages present in the NCBI databases. Contigs that contained contaminating host chromosomal DNA were discarded, and the contigs that entailed a full phage genome sequence were annotated using RASTtk with default parameters (14). Bacteriophage sequences were subsequently assigned to known species using two previously described multiplex PCR methods (15, 16). Fifteen and nine isolates could be assigned to the c2 and 936 species, respectively, using this approach. Following a previously proposed classification scheme (17), bacteriophages belonging to the c2 species were further classified into the subspecies c2 and bIL67. The analysis was conducted by comparing the C and N termini of their predicted receptor-binding proteins (RBP) (as shown by RASTtk annotation) and the complete sequences of the proteins encoded by the two adjacent open reading frames (ORFs) with those of the reference phages c2 and D4410. Based on the high (>80%) nucleotide similarity of their aforementioned ORFs to those of the two reference lactococcal bacteriophages, 12 isolates proved to belong to the c2 subspecies; the remaining 3 were shown to be part of the bIL67 subspecies. In cases in which no species-level results were obtained with the multiplex PCR approach, phage species were assigned via BLAST comparison of total genome sequences with publicly available lactococcal bacteriophage genomes. Four Bk5-T bacteriophages were identified in this way, based on >72% whole-genome similarity and a conserved genome organization, compared with known Bk5-T lactococcal bacteriophages.

The GC contents of the analyzed bacteriophages range from 34 to 36.4%. The genome lengths of the isolates range from 20 to 23.2 kb for the c2 phages, from 25.3 to 32.6 kb for phages of the 936 species, and from 25.3 to 32.6 kb for the Bk5-T members. The numbers of predicted ORFs range from 34 to 42 among members of the c2 species, from 46 to 62 among 936 phages, and from 51 to 60 for Bk5-T isolates.

### Data availability.

The complete genomic sequences of the 28 bacteriophages described here are available at GenBank under the accession numbers reported in Table 1. The SRA data for each genome reported here are available at the NCBI under BioProject accession number PRJNA606016.

**TABLE 1:** Characteristics of and accession numbers for the bacteriophages studied here

Bacteriophage name	Genome length (kb)	Sequencing coverage (1,000 fold)	No. of ORFs	Species^*a*^	GenBank accession no.	Host species	Host strain name
CHPC116	21.86	68.6	37	c2 (c2)	MN689507	*Lactococcus lactis* subsp. *lactis*	CH_LC20
CHPC122	22.1	67.8	41	c2 (c2)	MN689512	*Lactococcus lactis* subsp. *lactis*	CH_LC21
CHPC134	21.98	68.2	38	c2 (c2)	MN689515	*Lactococcus lactis* subsp. *cremoris*	CH_LC22
CHPC966	21.74	69	37	c2 (bIL67)	MN689526	*Lactococcus lactis* subsp. *lactis*	CH_LC23
CHPC967	22.4	67	42	c2 (c2)	MN689527	*Lactococcus lactis* subsp. *lactis*	CH_LC24
CHPC972	23.28	64.4	40	c2 (c2)	MN689528	*Lactococcus lactis* subsp. *lactis*	CH_LC25
CHPC973	22.3	67.2	36	c2 (c2)	MN689529	*Lactococcus lactis* subsp. *cremoris*	CH_LC26
CHPC1020	22.41	67	36	c2 (bIL67)	MN689505	*Lactococcus lactis* subsp. *lactis*	CH_LC27
CHPC1161	21.34	70.3	34	c2 (c2)	MN689506	*Lactococcus lactis* subsp. *cremoris*	CH_LC28
CHPC1170	21.74	69	40	c2 (c2)	MN689508	*Lactococcus lactis* subsp. *lactis*	CH_LC29
CHPC1182	20.75	72.3	34	c2 (bIL67)	MN689510	*Lactococcus lactis* subsp. *lactis*	CH_LC30
CHPC1183	20.03	74.8	40	c2 (c2)	MN689511	*Lactococcus lactis* subsp. *cremoris*	CH_LC31
CHPC1242	21.14	71	35	c2 (c2)	MN689513	*Lactococcus lactis* subsp. *lactis*	CH_LC32
5171F	21.07	71.2	37	c2 (c2)	MN689503	*Lactococcus lactis* subsp. *cremoris*	MG1363
5205F	21.21	70.7	35	c2 (c2)	MN689504	*Lactococcus lactis* subsp. *cremoris*	MG1363
CHPC52	29.65	50.6	54	936	MN689519	*Lactococcus lactis* subsp. *cremoris*	CH_LC33
CHPC129	30.83	48.6	55	936	MN689514	*Lactococcus lactis* subsp. *cremoris*	CH_LC34
CHPC361	30.16	49.7	55	936	MN689517	*Lactococcus lactis* subsp. *cremoris*	CH_LC35
CHPC362	27.64	54.2	46	936	MN689518	*Lactococcus lactis* subsp. *cremoris*	CH_LC36
CHPC781	29.21	51.3	56	936	MN689520	*Lactococcus lactis* subsp. *cremoris*	CH_LC37
CHPC958	32.65	45.5	62	936	MN689522	*Lactococcus lactis* subsp. *cremoris*	CH_LC38
CHPC959	29.34	51.1	59	936	MN689523	*Lactococcus lactis* subsp. *cremoris*	CH_LC39
CHPC964	29.92	50.1	56	936	MN689524	*Lactococcus lactis* subsp. *cremoris*	CH_LC40
CHPC965	25.32	59.2	47	936	MN689525	*Lactococcus lactis* subsp. *cremoris*	CH_LC41
CHPC148	33.55	44.7	51	BK5-T	MN689516	*Lactococcus lactis* subsp. *lactis*	CH_LC42
CHPC836	36.48	41.1	57	BK5-T	MN689521	*Lactococcus lactis* subsp. *lactis*	CH_LC43
CHPC974	33.79	44.4	60	BK5-T	MN689530	*Lactococcus lactis* subsp. *lactis*	CH_LC44
CHPC1175	36.33	41.3	52	BK5-T	MN689509	*Lactococcus lactis* subsp. *lactis*	CH_LC45

a The subspecies of c2 bacteriophages is given.
